# Liver-specific lncRNA FAM99A may be a tumor suppressor and promising prognostic biomarker in hepatocellular carcinoma

**DOI:** 10.1186/s12885-022-10186-2

**Published:** 2022-10-26

**Authors:** Meile Mo, Xiaoyun Ma, Yihuan Luo, Chao Tan, Bihu Liu, Peng Tang, Qian Liao, Shun Liu, Hongping Yu, Dongping Huang, Xiaoyun Zeng, Xiaoqiang Qiu

**Affiliations:** 1 Department of Epidemiology, School of Public Health, Guangxi Medical University, Nanning, Guangxi 530021 P.R. China; 2 Department of Epidemiology, School of Public Health, Cheeloo College of Medicine, Shandong University, Jinan, Shandong 250012 P.R. China; 3 Department of Acute Care Surgery, The First Affiliated Hospital of Guangxi Medical University, Nanning, Guangxi 530021 P.R. China; 4 Department of Epidemiology and Statistics, School of Public Health, Guilin Medical University, Guilin, Guangxi 541004 P.R. China; 5 Department of Maternal, Child and Adolescent Health, School of Public Health, Guangxi Medical University, Nanning, Guangxi 530021 P.R. China; 6 Guangxi Medical University Cancer Hospital, Nanning, Guangxi 530021 P.R. China; 7 Department of Sanitary Chemistry, School of Public Health, Guangxi Medical University, Nanning, Guangxi 530021 P.R. China

**Keywords:** Hepatocellular carcinoma, Liver-specific lncRNA, FAM99A, Prognosis, Tumor growth

## Abstract

**Background:**

Increasing evidence shows that liver-specific long non-coding RNAs (lncRNAs) play important roles in the development of hepatocellular carcinoma (HCC). We identified a novel liver-specific lncRNA, FAM99A, and examined its clinical significance and biological functions in HCC.

**Methods:**

The expression level and clinical value of FAM99A in HCC were examined using The Cancer Genome Atlas (TCGA), International Cancer Genome Consortium (ICGC), and Gene Expression Omnibus (GEO) databases, and were further verified using quantitative real-time polymerase chain reaction (qRT–PCR) in our HCC cohort. Univariate and multivariate Cox proportional hazards regression models were also applied to identify independent prognostic indicators for HCC patients. Cell counting kit-8, colony formation, and Transwell assays were performed to evaluate the effects of FAM99A on the proliferation, migration, and invasion abilities of HCC cells in vitro. A subcutaneous xenograft tumor model was implemented to determine the effect of FAM99A on the tumor growth of HCC cells in vivo. RNA pull-down and mass spectrometry assays were performed to reveal the potential molecular mechanisms of FAM99A in HCC.

**Results:**

The three public online databases and qRT–PCR data showed that FAM99A was frequently downregulated in HCC tissues and inversely correlated with microvascular invasion and advanced histological grade of HCC patients. Kaplan–Meier survival analysis indicated that decreased FAM99A was significantly associated with poor overall survival of HCC patients based on TCGA database (*P* = 0.040), ICGC data portal (*P* < 0.001), and our HCC cohort (*P* = 0.010). A multivariate Cox proportional hazards regression model based on our HCC cohort suggested that FAM99A was an independent prognostic factor of overall survival for HCC patients (hazard ratio: 0.425, *P* = 0.039). Upregulation of FAM99A suppressed the proliferation, colony formation, migration, and invasion capacities of HCC cells in vitro, and knockdown of FAM99A had the opposite effects. A subcutaneous xenograft tumor model demonstrated that overexpression of FAM99A significantly inhibited the tumor growth of HCC cells in vivo. Seven tumor-related proteins (PCBP1, SRSF5, SRSF6, YBX1, IGF2BP2, HNRNPK, and HNRNPL) were recognized as possible FAM99A-binding proteins by the RNA pull-down and mass spectrometry assays.

**Conclusion:**

Our results suggest that FAM99A exerts cancer-inhibiting effects on HCC progression, and it may be a promising prognostic indicator for HCC patients.

**Supplementary Information:**

The online version contains supplementary material available at 10.1186/s12885-022-10186-2.

## Introduction

Liver cancer is a common solid malignancy. It was the sixth most common tumor and the third leading cause of cancer-related mortality worldwide according to the World Health Organization’s statistics in 2020 [1]. China has a high incidence of liver cancer, which account for more than half of all cases worldwide [2]. For Chinese males, liver cancer ranks second only to lung cancer in cancer-related deaths [3]. Of all types of liver cancer, hepatocellular carcinoma (HCC) is the most common type, and it is responsible for approximately 75-80% of all liver cancers. Hepatic resection and liver transplantation remain the first choice for patients with early- and mid-stage HCC. However, the 5-year recurrence rate of HCC is approximately 80% after radical treatment [4]. Most patients are diagnosed with late-stage HCC, who have limited therapeutic options and generally have expected median survival times of 6-8 months [5]. Therefore, more research is urgently needed to comprehensively understand the molecular mechanisms of HCC and identify new biomarkers and therapeutics for HCC patients.

Long non-coding RNAs (lncRNAs) are a class of endogenous non-coding RNAs that are longer than 200 nucleotides and exert pivotal effects in the pathogenesis and progression of cancers [6–8]. Accumulating studies have shown that lncRNAs play important roles in regulating various biological processes, including chromatin and genome dynamics, gene expression, development, and cell differentiation [9–11]. Many dysregulated lncRNAs are involved in the tumorigenesis and development of HCC. For example, lncRNA PCNAP1 accelerated hepatitis B virus (HBV) replication and hepatocarcinogenesis by modulating the miR-154/PCNA/HBV cccDNA signaling pathway [12]. LINC00662 was upregulated in HCC and promoted HCC progression by activating the Wnt/β-catenin signaling pathway and M2 macrophage polarization [13]. Another lncRNA, p53-stabilizing and activating RNA (PSTAR), was downregulated in HCC and suppressed HCC cell proliferation and tumorigenicity by inducing p53-mediated cell cycle arrest [14]. However, the mechanisms of HCC are not clear, and many dysregulated lncRNAs must be explored.

Compared to protein-coding genes, lncRNAs have more tissue-specific expression characteristics [6]. Many lncRNAs with liver-specific expression patterns were identified as significantly related to the occurrence and progression of HCC. For example, lncRNA HULC (highly upregulated in liver cancer) accelerates HCC progression and attenuates the sensitivity of HCC cells to chemotherapeutic agents [15–18]. LINC01093 is downregulated in HCC tissues and suppresses HCC growth and metastasis [19]. Our previous studies also identified three liver-specific lncRNAs, FAM99B [20], LINC02499 [21], and LINC01146 [22], that were all downregulated in HCC and exerted similar inhibitory effects on the proliferation, migration, and invasion of HCC cells.

The current study identified a novel liver-specific lncRNA, FAM99A (family with sequence similarity 99 member A), which was specifically expressed in normal liver tissues based on the RNA-sequencing data from the Genotype-Tissue Expression (GTEx) project (https://gtexportal.org/home/) (Additional file 1: Figure S1). Given the great importance of liver-specific lncRNAs in the development of HCC, we comprehensively examined the expression level of FAM99A in HCC tissues based on public online databases and our HCC cohort. The clinical significance and prognostic value of FAM99A in HCC patients were also investigated. The biological function of FAM99A on HCC cell proliferation, migration, invasion, and tumor growth was evaluated in vitro and in vivo. RNA pull-down assay and mass spectrometry analysis were also performed to investigate the potential molecular mechanisms of FAM99A in impeding the progression of HCC.

## Methods

### Human tissue specimens

The Ethics Committee of Guangxi Medical University approved the study. HCC tissues and corresponding paracancerous tissues were collected from 62 HCC patients who underwent radical surgical resection at the Affiliated Cancer Hospital of Guangxi Medical University between February 2016 and December 2019. The diagnosis of HCC was confirmed by pathological examination. The tissue samples were snap frozen in liquid nitrogen and stored in liquid nitrogen until use. Patients with a history of preoperative chemotherapy or radiotherapy were excluded. Informed consent was obtained from all included patients, and patients were followed up until December 2021.

### Public online databases

The RNA sequencing (RNA-seq) data (371 HCC tissues and 50 adjacent normal tissues) of HCC patients (level 3) were extracted from The Cancer Genome Atlas (TCGA) database (https://cancergenome.nih.gov/) (up to January 14, 2019). Transcript expression data were calculated as transcripts per million (TPM) and normalized by converting to log2 (TPM + 1). The clinical parameters and follow-up information were also downloaded to assess the clinical significance of FAM99A. For overall survival (OS) analysis, 370 cases were included after eliminating one patient without follow-up OS data.

The RNA-seq and clinical data of another HCC cohort were obtained from the International Cancer Genome Consortium (ICGC) data portal (https://dcc.icgc.org/) (Data Release 28) on June 2, 2020. Due to a lack of paracancerous tissue expression data, the Liver Cancer, France [LICA-FR] cohort was excluded from our study. For the Liver Cancer, RIKEN, Japan [LIRI-JP] cohort, a total of 221 HCC tissues and 200 adjacent normal tissues were enrolled in the research after eliminating two metastatic tumor cases. The normalized read counts in this cohort were used for analysis, and OS analysis was performed to evaluate the prognostic significance of FAM99A.

To draw a comprehensive result, we also extracted microarray datasets containing FAM99A expression data from the Gene Expression Omnibus (GEO) database (http://www.ncbi.nlm.nih.gov/geo/) (up to October 4, 2020). The retrieved keywords are listed below: (long non-coding RNAs OR lncRNAs OR non-coding RNAs) AND (hepatocellular carcinoma OR HCC OR liver) AND (cancer OR tumor OR carcinoma OR neoplasm* OR malignant*). The “Top Organisms” was restricted to “Homo sapiens”. Studies that simultaneously met the following two inclusion criteria were included: 1) HCC tissues and peritumoral liver specimens were included in the study (≥ 5 samples each group); and 2) the RNA profiling included the expression data of FAM99A. The basic information and FAM99A expression were carefully extracted from eligible datasets. A meta-analysis based on GEO datasets was subsequently performed.

### Cell culture and cell transfection

The human HCC cell lines (Huh-7, Hep 3B, HepG2, HCCLM3, MHCC97L and MHCC97H) were maintained in our laboratory, and their background information has been previously described in detail [20]. Cells were cultured in Dulbecco’s modified Eagle’s medium (DMEM, Gibco, USA) or Minimum Essential Medium (MEM, Gibco, USA) supplemented with 10% fetal bovine serum (FBS, Gibco, Australia) at 37 °C in a humidified incubator containing 5% CO_2_.

To obtain FAM99A overexpression cell lines, full-length FAM99A (1430 bp) was ligated into the LV5 (EF-1aF/GFP&Puro) vector. A nonsense oligonucleotide was used as a negative control (Lv-NC). The lentiviruses were synthesized by GenePharma Co., Ltd. (Shanghai, China). For FAM99A knockdown, short hairpin RNA (shRNA) targeting FAM99A was inserted into the GV493 (hU6-MCS-CBh-gcGFP-IRES-puromycin) vector. A non-silencing shRNA was used as a negative control (sh-NC). The lentiviruses were synthesized by Genechem Co., Ltd. (Shanghai, China). The following shRNA sequences were used: sh-NC, 5’-TTCTCCGAACGTGTCACGT-3’; sh-FAM99A, 5’-AATAAAAGTCACAGGACAA-3’. Cells (8 × 10^4^ cells/well) were seeded into 6-well plates, incubated for 24 hours, then infected with lentiviruses according to the manufacturer’s instructions. Seventy-two hours after infection, the cells were exposed to puromycin (3.5 μg/ml) for two weeks to obtain stably transfected cell lines. The overexpression and knockdown efficiency of FAM99A in HCC cell lines were verified using qRT-PCR.

### RNA extraction, reverse transcription, and qRT-PCR

Total RNA of liver cancer cells and HCC samples was extracted using TRIzol reagent (Invitrogen, USA), and 900 ng of total RNA was reverse transcribed to complementary DNA using the PrimeScript™ RT reagent Kit with gDNA Eraser (Takara, Japan). The relative RNA expression level was evaluated using the TB GreenTM Premix Ex TaqTM II Kit (Takara, Japan) in a real-time PCR system (Applied Biosystems StepOnePlus, USA). GAPDH was used as an internal control. The transcript level was calculated using the 2^−△△Ct^ method, and the primers for GAPDH and FAM99A are listed below:

GAPDH-forward: 5’-AGCCACATCGCTCAGACAC-3’,

GAPDH-reverse: 5’-GCCCAATACGACCAAATCC-3’;

FAM99A-forward: 5’-CTCTTGTCCAGGTCAGCATCTC-3’,

FAM99A-reverse: 5’-ACGCATCACAAAACAGCCAC-3’.

### Subcellular fractionation analysis

Isolation of cytoplasmic and nuclear RNAs in Hep 3B cells was performed using the PARIS Kit (Invitrogen, USA) according to the manufacturer’s instructions. The relative expression of FAM99A in cytoplasmic and nuclear fractions was determined using qRT-PCR. GAPDH and U6 were used as cytoplasmic and nuclear controls, respectively. The following primers were used for U6: U6-forward: 5’-CGCTTCGGCAGCACATATA-3’; U6-reverse: 5’-TTCACGAATTTGCGTGTCAT-3’.

### Cell viability and colony formation assays

For the Cell Counting Kit-8 (CCK-8, Dojindo, Japan) cell proliferation viability assay, stably transfected Huh-7 (3000 cells/well) and Hep 3B (2000 cells/well) cells were seeded into 96-well plates. A total of 10 μl of CCK-8 reagent was added to each well after 1, 2, 3, 4, and 5 days of incubation, and the absorbance was measured at 450 nm after incubation at 37 °C for another 2 hours. For the plate clone formation assay, stably transfected Huh-7 (1500 cells/well) and Hep 3B (500 cells/well) cells were seeded into 6-well plates and maintained in medium supplementing with 10% FBS for 14 days (Huh-7) or 10 days (Hep 3B). Colonies were fixed with methanol for 15 minutes, and stained with 0.1% crystal violet for another 15 minutes.

### Cell migration and invasion assays

Transwell inserts (Costar, Corning, USA) with 8-μm polycarbonate membranes were used to assess the migration ability of cancer cells. The upper chambers coated with Matrigel (Corning, USA) were used for the invasion assay in vitro. Briefly, 200 μl serum-free medium containing 1×10^5^ cells was added to the upper chambers. The bottom chambers received 600 μl culture medium containing 10% FBS. After incubating for 1 day (Huh-7) or 2 days (Hep 3B), the cells remaining in the upper chambers were gently wiped with wet cotton swabs. The migrated or invaded cells were fixed with methanol and stained with 0.1% crystal violet for 15 minutes. Five fields were randomly selected for imaging using the microscope *EVOS FL Auto* Cell Imaging System (*EVOS FL*, Thermo Fisher Scientific, USA).

### In vivo tumorigenesis experiments

BALB/c nude mice (4 weeks, male) were provided by the Experimental Animal Center of Guangxi Medical University and raised under specific pathogen free (SPF) conditions. Nude mice were randomly divided into 2 groups (n = 8 per group). A total of 5×10^6^ Huh-7 cells stably transfected with FAM99A overexpression (Lv-FAM99A) or negative control (Lv-NC) were resuspended in a mixture of 50 μl PBS and 50 μl Matrigel (Corning, USA) and subcutaneously injected into the right flank of mice. The tumor size was measured every 3 days using a Vernier Caliper, and the tumor volume was calculated by the formula: tumor volume (mm^3^) = 0.5×L×W^2^ (L, longest diameter; W: shortest diameter). Four weeks later, the nude mice were sacrificed, and their subcutaneous tumors were isolated and weighed. The tumors were divided into two parts. One part was frozen in liquid nitrogen for RNA extraction, and the other part was fixed using 4% paraformaldehyde for hematoxylin-eosin (HE) and immunohistochemical (IHC) staining. All animal experiments were performed in accordance with the Guiding Principles for Care and Use of Experimental Animals, and the Animal Care & Welfare Committee of Guangxi Medical University approved this study (approval number: 202012016).

### HE & IHC

Paraformaldehyde-fixed tissues were trimmed, dehydrated in gradient alcohol and embedded in paraffin. After deparaffinization with xylene and rehydration in an ethanol gradient, paraffin sections were stained with hematoxylin for 5~10 minutes followed by several dips in 1% hydrochloric acid alcohol. After rinsing with distilled water, the sections were stained with a 1% eosin aqueous solution for 3 minutes, dehydrated in gradient alcohol and cleared with xylene.

For IHC staining, citrate buffer was used for antigen retrieval, and 3% hydrogen peroxide was used to block the activity of endogenous peroxidase. The paraffin sections were washed with PBS and incubated with the diluted primary antibody anti-Ki67 (1:200, Abcam, ab16667) for 1 hour at 37 °C. After incubation with horseradish peroxidase (HRP)-labeled secondary antibody for 30 minutes, sections were stained with diaminobenzidine (DAB) chromogen and counterstained with hematoxylin. The sections were scanned using the *TissueFAXS PLUS* system (TissueGnostic, Austria), and the sum of integrated optical density (IOD) was calculated using *Image-Pro Plus 6.0* software.

### RNA fluorescence in situ hybridization (RNA-FISH)

Cy3-labeled oligonucleotide probes specifically targeting FAM99A were designed and synthesized by Genechem (Shanghai, China). Human 18S FISH probe mix and U6 FISH probe mix were synthesized by RiboBio (Guangzhou, China). RNA-FISH was accomplished using a Ribo^TM^ Fluorescent in Situ Hybridization Kit (RiboBio, Guangzhou, China) according to the manufacturer’s protocol. Briefly, Hep 3B and Huh-7 cells were cultured on cell climbing slices, washed with PBS, and fixed with 4% paraformaldehyde for 10 minutes. After washing with PBS three times, the cells were permeabilized with 0.5% Triton X-100 for 5 minutes at 4 °C. The permeabilized cells were incubated with pre-hybridization buffer for 30 minutes and incubated with hybridization buffer containing probes overnight at 37 °C. Nuclei were counterstained with 4′-6-diamidino-2-phenylindole (DAPI) for 10 minutes, and cells were washed with PBS three times. Cells were visualized, and images were captured using a confocal microscope (*LSM 800*, Zeiss, Germany).

### RNA pull-down assay and mass spectrometry analysis

RNA pull-down was performed using the Pierce Magnetic RNA-Protein Pull-Down Kit (Thermo Fisher Scientific, USA). The sense and antisense chains of FAM99A were transcribed in vitro using T7 RNA Polymerase (Roche, USA), and labeled with Biotin RNA Labeling Mix (Roche, USA) according to the manufacturer’s instructions. The biotin-labeled RNAs were incubated with streptavidin magnetic beads (Invitrogen, USA) at 4 °C overnight to obtain the bead-RNA complex. Cell lysates (approximately 1 mg protein) were added to the bead-RNA complexes and incubated at room temperature for 1 hour. The precipitated complexes were washed with washing buffer three times and boiled in SDS buffer. The retrieved RNA-binding proteins were separated using electrophoresis and visualized by silver staining. The eluted proteins were also collected to performed mass spectrometry analysis using a *Q Exactive System* (Thermo Fisher Scientific, USA).

### Statistical analysis

Numerical data are presented as the means ± standard deviation (SD). Paired-samples and independent-samples *t* tests were implemented to compare differences between two groups. The expression level of FAM99A was divided into high expression and low expression groups according to their median values. The *chi-squared* test was performed to explore the clinicopathological significance of FAM99A. The OS between the high and low FAM99A expression groups was compared using Kaplan–Meier curves with the log-rank test (two sides). Univariate and multivariate Cox proportional hazards regression models were also applied to determine the independent prognostic factors of HCC patients. All of the above analyses were performed using *SPSS 20.0* software, and the results with *P* < 0.05 were considered statistically significant. The graphs were drawn using *GraphPad Prism 8.0* software.

To integrate the microarray results of GEO datasets, meta-analysis was implemented using *STATA 15.0* software. The *chi-squared* and *I-squared* tests were used to examine the heterogeneity between the included datasets. When *I*^*2*^ > 50% or *P* < 0.05, significant heterogeneity existed among datasets, and the random-effect model was selected. Otherwise, the fixed-effect model was used for meta-analysis. A forest plot was drawn to obtain the standardized mean difference (*SMD*) and 95% confidence interval (*CI*). When *P* < 0.05, a pooled *SMD* < 0 indicated that the expression level of FAM99A was significantly downregulated in HCC tissues compared to adjacent normal tissues. In contrast, FAM99A was considered upregulated in HCC tissues when *P* < 0.005 and the combined *SMD* > 0.

## Results

### FAM99A is downregulated in HCC and negatively correlated with poor prognosis of HCC patients based on TCGA and ICGC databases

**Fig. 1:**
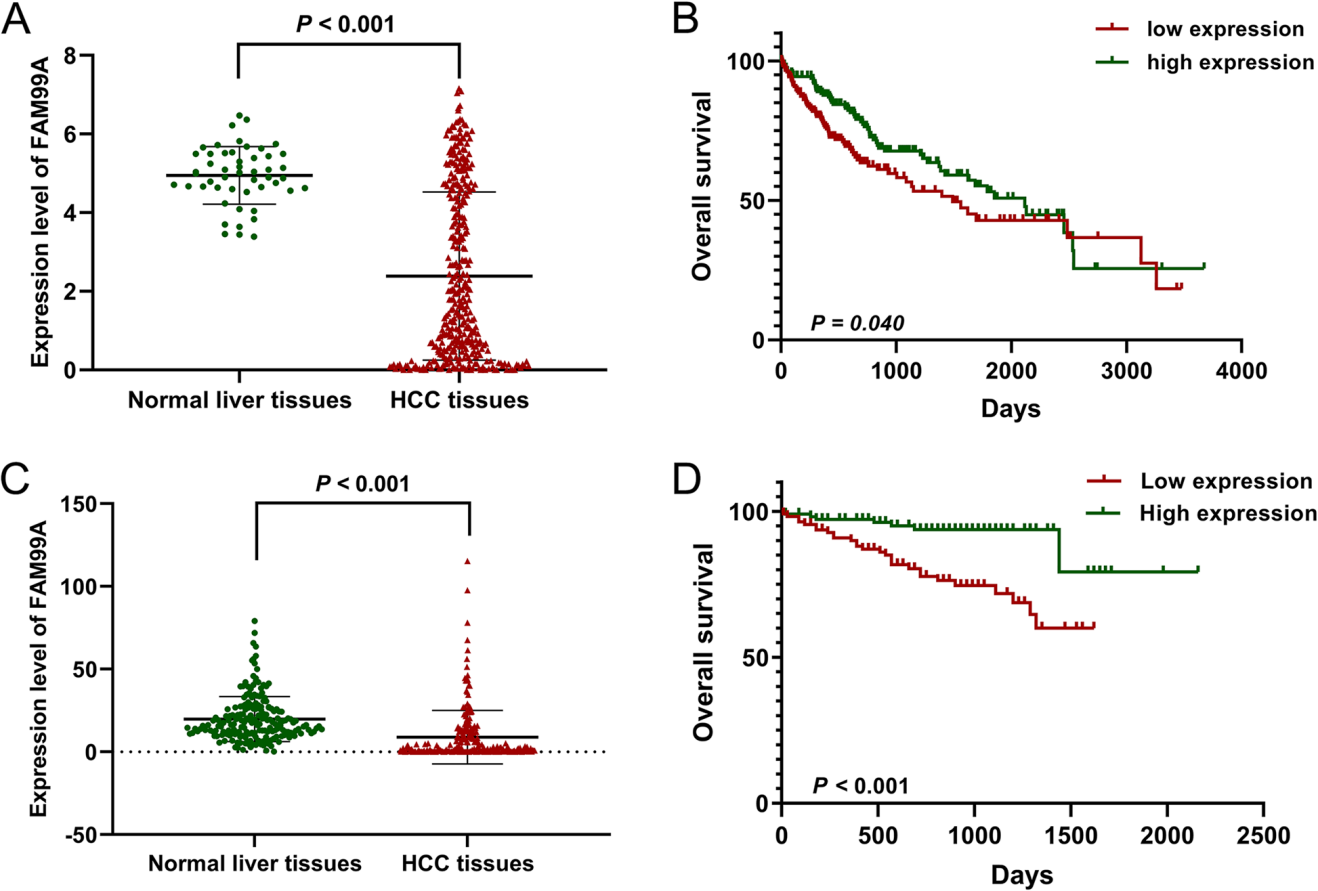
FAM99A is frequently downregulated in HCC tissues based on public online databases. **A** The expression level of FAM99A in 371 HCC tissues and 50 noncancerous tissues based on TCGA database. **B** Kaplan-Meier analysis showed that low expression of FAM99A was related to poor overall survival of HCC patients in TCGA database (n = 370). **C** The expression level of FAM99A in 221 HCC tissues and 200 adjacent normal tissues based on the ICGC data portal. **D** Kaplan-Meier analysis revealed that downregulated expression of FAM99A remarkably correlated with shorter overall survival of HCC patients in the ICGC data portal (n = 221)

To investigate the role of FAM99A in HCC development, we extracted the RNA-seq data of FAM99A from TCGA database. The results indicated that the expression level of FAM99A in HCC tissues was significantly lower than noncancerous tissues (2.39 ± 2.14 vs. 4.95 ± 0.73; *P* < 0.001; Fig. 1A). The patients were divided into high (> 1.619) and low expression (≤ 1.619) groups according to the median value of FAM99A. The clinical significance of FAM99A in HCC patients was also examined. The results revealed that low FAM99A expression significantly correlated with vascular invasion (*P* < 0.001) and advanced histological grade (*P* = 0.004; Table 1). The Kaplan–Meier survival curve showed that HCC patients with lower FAM99A expression tended to have poor OS (*χ*^*2*^ = 4.199, *P* = 0.040; Fig. 1B).

**Table 1:** The relationship between FAM99A expression and clinical parameters in TCGA dataset

Clinical parameters	N	FAM99A expression		χ^**2**^	***P***
		Low expression	High expression		
Age^a^ (years)				1.548	0.213
≤ 60	177	94	83		
> 60	193	90	103		
Gender				0.006	0.941
Male	250	125	125		
Female	121	60	61		
Race^a^				1.286	0.257
Asian	158	85	73		
Non-Asian	203	97	106		
Hepatitis B virus infection^a^				0.367	0.545
Negative	248	120	128		
Positive	104	54	50		
Hepatitis C virus infection^a^				2.743	0.098
Negative	296	152	144		
Positive	56	22	34		
Vascular invasion^a^				15.688	**< 0.001**
None	206	87	119		
Microvascular invasion	93	59	34		
Macrovascular invasion	16	12	4		
Histological grade^a^				8.413	**0.004**
G1-G2	232	102	130		
G3-G4	134	80	54		
Pathological stage^a^				1.719	0.190
I-II	257	125	132		
III-IV	90	51	39		
T stage^a^				1.166	0.280
I-II	275	133	142		
III-IV	93	51	42		
N stage^a^				0.168	0.682
N0	252	131	121		
N1	4	3	1		
M stage^a^				0.184	0.668
M0	266	137	129		
M1	4	3	1		
Child-Pugh classification grade ^a^				3.430	0.064
A	217	103	114		
B+C	22	15	7		

Statistically significant *P* values are in bold (*P* < 0.05)

*Macro/micro* Macrovascular invasion/microvascular invasion; *TNM* staging system according to the American Joint Committee on Cancer (AJCC) with T tumor, N lymph nodes metastasis, M distant metastasis

^a^ Represent have missing value

We also examined FAM99A expression in the LIRI-JP cohort based on the ICGC database. The results showed that FAM99A was downregulated in HCC tissues versus adjacent normal tissues (8.93 ± 16.21 vs. 19.83 ± 13.65; *P* < 0.001; Fig. 1C). To examine the prognostic significance of FAM99A, 221 patients were divided into high and low expression groups based on the median (2.00) of FAM99A. Kaplan–Meier survival analysis demonstrated that patients in the low FAM99A expression group had a shorter OS time than patients in the high FAM99A expression group (*χ*^*2*^ = 13.495, *P* < 0.001; Fig. 1D).

### Meta-analysis of FAM99A expression in HCC based on the GEO database

**Fig. 2:**
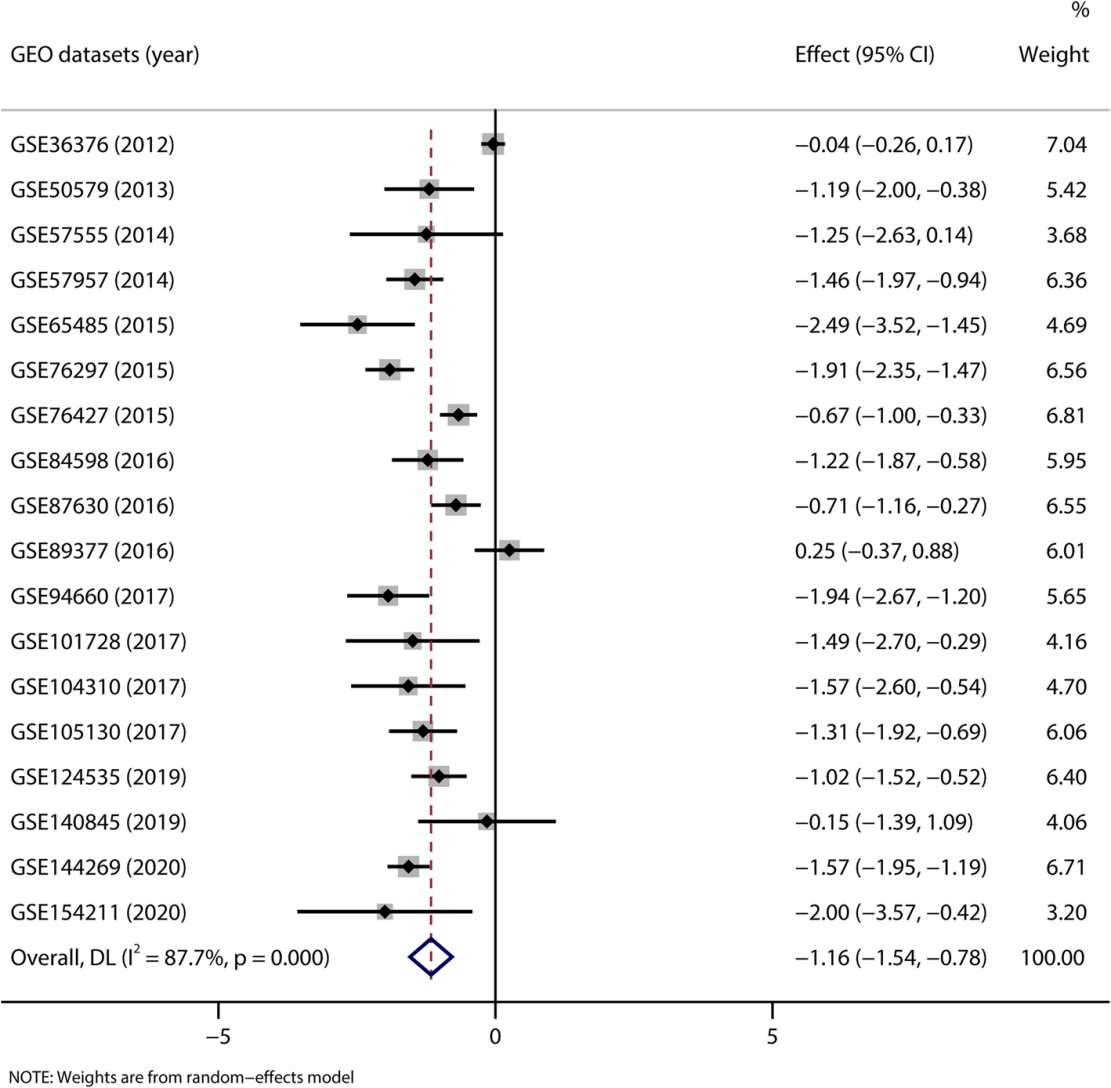
A forest plot of 18 GEO datasets for the FAM99A expression level in HCC tissues (using a random-effect model)

To support a comprehensive conclusion, we also retrieved microarray chip data containing FAM99A expression from the GEO database. Based on our inclusion criteria, 18 eligible GEO datasets were enrolled, and detailed information of these GEO datasets is listed in Table 2. As shown in Fig. 2, there was significant heterogeneity among these 18 GEO datasets (*I*^*2*^ = 87.7%; *P* < 0.001). Therefore, a random-effect model was applied for the meta-analysis. The pooled *SMD* suggested that the expression of FAM99A was decreased in HCC tissues compared to noncancerous tissues (*SMD* = -1.162, 95% *CI* (-1.541, -0.783); *P* < 0.001; Fig. 2).

**Table 2:** The basic information and FAM99A expression level of included GEO datasets

GEO datasets	Year	Platform	Country/Region	Tissue types	N	FAM99A expression	***t***	***P***
						(Mean ± SD)		
GSE36376	2012	GPL10558	South Korea	HCC tissue	240	6.68 ± 0.24	-0.484	0.629
				Normal tissue	130	6.69 ± 0.22		
GSE50579	2013	GPL14550	Germany	HCC tissue	60	9.43 ± 3.56	-7.461	**< 0.001**
				Normal tissue	7	13.49 ± 0.77		
GSE57555	2014	GPL16699	Japan	HCC tissue	5	-0.03 ± 0.26	-2.595	0.060
				Normal tissue	5	0.23 ± 0.14		
GSE57957	2014	GPL10558	Singapore	HCC tissue	37	8.00 ± 0.53	-8.095	**< 0.001**
				Normal tissue	37	8.75 ± 0.50		
GSE65485	2015	GPL11154	China	HCC tissue	50	1.77 ± 1.43	-5.302	**< 0.001**
				Normal tissue	5	5.21 ± 0.52		
GSE76297	2015	GPL17586	USA	HCC tissue	58	6.11 ± 0.66	-11.390	**< 0.001**
				Normal tissue	58	7.13 ± 0.37		
GSE76427	2015	GPL10558	Singapore	HCC tissue	115	7.85 ± 0.87	-3.948	**< 0.001**
				Normal tissue	52	8.40 ± 0.72		
GSE84598	2016	GPL10558	Germany	HCC tissue	22	4.42 ± 2.75	-4.165	**< 0.001**
				Normal tissue	22	7.00 ± 1.16		
GSE87630	2016	GPL6947	South Korea	HCC tissue	64	7.61 ± 1.02	-3.928	**< 0.001**
				Normal tissue	30	8.25 ± 0.55		
GSE89377	2016	GPL6947	South Korea	HCC tissue	40	7.49 ± 0.96	1.096	0.279
				Normal tissue	13	7.27 ± 0.47		
GSE94660	2017	GPL16791	USA	HCC tissue	21	1.92 ± 1.62	-6.052	**< 0.001**
				Normal tissue	21	4.33 ± 0.69		
GSE101728	2017	GPL21047	China	HCC tissue	7	9.08 ± 3.63	-2.790	**0.032**
				Normal tissue	7	12.96 ± 0.57		
GSE104310	2017	GPL16791	China	HCC tissue	12	1.51 ± 1.83	-3.991	**0.001**
				Normal tissue	8	3.88 ± 0.76		
GSE105130	2017	GPL11154	Singapore	HCC tissue	25	1.74 ± 1.70	4.587	**< 0.001**
				Normal tissue	25	3.46 ± 0.76		
GSE124535	2019	GPL20795	China	HCC tissue	35	1.44 ± 1.22	-5.099	**< 0.001**
				Normal tissue	35	2.59 ± 1.03		
GSE140845	2019	GPL16791	India	HCC tissue	5	4.16 ± 2.08	-0.241	0.816
				Normal tissue	5	4.44 ± 1.56		
GSE144269	2020	GPL24676	USA	HCC tissue	70	1.16 ± 3.79	-9.142	**< 0.001**
				Normal tissue	70	5.56 ± 1.18		
GSE154211	2020	GPL11154	Taiwan	HCC tissue	5	0.53 ± 0.45	-3.184	**0.033**
				Normal tissue	5	2.22 ± 1.11		

Statistically significant *P* values are in bold (*P* < 0.05)

*GEO* Gene Expression Omnibus; *HCC* hepatocellular carcinoma; *SD* standard deviation

### The decreased expression and clinical significance of FAM99A in HCC were verified based on our HCC cohort

**Fig. 3:**
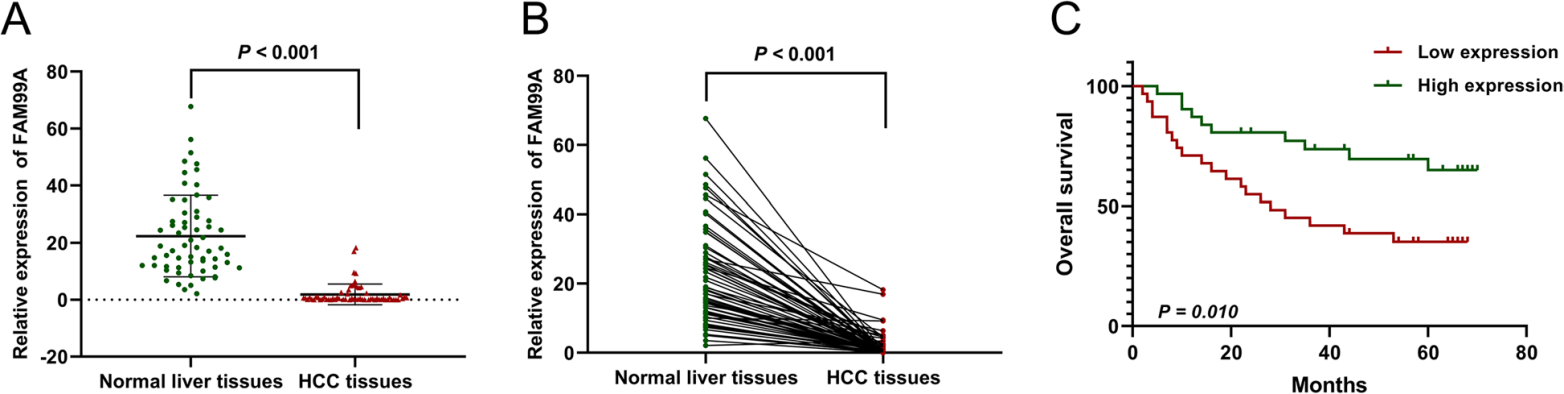
The decreased expression of FAM99A in HCC was verified based on our HCC cohort. **A**, **B** Relative expression level of FAM99A measured using qRT-PCR in 62 pairs of HCC tissues and corresponding adjacent normal tissues. **C** Kaplan–Meier analysis demonstrated that decreased FAM99A expression was remarkably associated with reduced overall survival of HCC patients in our cohort (n = 62)

To further confirm the expression level of FAM99A in HCC, qRT-PCR was performed to compare FAM99A expression between 62 pairs of HCC and corresponding peritumoral liver specimens. We found that FAM99A was remarkably downregulated in 98.39% (61/62) of HCC samples (1.84 ± 3.62 vs. 22.27 ± 14.27; *P* < 0.001; Fig. 3A, B). To investigate the clinical significance of FAM99A in HCC, patients were also divided into a high expression group (> 0.417) and a low expression group (≤ 0.417) on the basis of the median expression of FAM99A. Consistent with the TCGA results, low expression of FAM99A significantly correlated with microvascular invasion (*P* = 0.041; Table 3).

**Table 3:** Correlation of FAM99A expression with clinicopathologic features of patients in our HCC cohort.

Clinicopathological features	N	FAM99A expression		χ^**2**^	***P***
		Low expression	High expression		
Age (years)				1.033	0.309
≤ 50	30	13	17		
> 50	32	18	14		
Gender				0.000	1.000
Male	52	26	26		
Female	10	5	5		
Liver cirrhosis				0.295	0.587
Negative	20	11	9		
Positive	42	20	22		
Hepatitis B virus infection				0.144	0.705
Negative	8	3	5		
Positive	54	28	26		
AFP level (ng/ml)				1.033	0.309
≤ 400	32	18	14		
> 400	30	13	17		
Tumor size (cm)				3.161	0.075
< 7	31	12	19		
≥ 7	31	19	12		
Tumor number				0.477	0.490
Single	52	25	27		
Multiple	10	6	4		
Microvascular invasion				4.168	**0.041**
Negative	28	10	18		
Positive	34	21	13		
Lymph nodes metastasis				0.000	1.000
Negative	58	29	29		
Positive	4	2	2		
Edmondson-Steiner grade				0.265	0.607
I-II	26	12	14		
III-IV	36	19	17		
BCLC stage				4.633	0.099
A stage	31	12	19		
B stage	12	9	3		
C stage	19	10	9		
Child-Pugh classification grade				0.000	1.000
A	57	28	29		
B	5	3	2		

Statistically significant *P* values are in bold (*P* < 0.05)

*HCC* hepatocellular carcinoma; *AFP* alpha-fetoprotein; *BCLC* Barcelona Clinic Liver Cancer

Kaplan–Meier survival curve was also performed, and the results showed that decreased FAM99A expression was remarkably associated with reduced OS of patients in our cohort (*χ*^*2*^ = 6.658, *P* = 0.010; Fig. 3C). The results of univariate Cox regression analysis found that tumor size, BCLC stage, Child-Pugh classification grade, microvascular invasion, Edmondson-Steiner grade, and FAM99A expression level correlated with the OS of HCC patients (*P* < 0.1; Table 4). Further multivariate Cox regression analysis demonstrated that the expression level of FAM99A was an independent prognostic factor for HCC patients (hazard ratio: 0.425, 95% CI: 0.189–0.958, *P* = 0.039; Table 4).

**Table 4:** Univariate and multivariate Cox proportional hazards regression models of overall survival in 62 HCC patients

Clinicopathological features	Univariate analysis				Multivariable analysis			
	***β***	HR	95%CI	***P***	***β***	HR	95%CI	***P***
Age (≥ 50 years vs.< 50 years)	0.274	1.315	0.638-2.712	0.458				
Sex (female vs. male)	0.101	1.107	0.423-2.892	0.836				
Liver cirrhosis (positive vs. negative)	0.528	1.695	0.726-3.956	0.222				
Hepatitis B virus infection (positive vs. negative)	-0.327	0.721	0.275-1.890	0.506				
AFP (> 400 ng/ml vs. ≤ 400 ng/ml)	0.173	1.188	0.580-2.435	0.637				
Tumor number (multiple vs. single)	0.631	1.880	0.806-4.386	0.144				
Lymph nodes metastasis (positive vs. negative)	0.938	2.555	0.765-8.539	0.127				
Tumor size (≥ 7cm vs. < 7cm)	0.802	2.229	1.059-4.691	**0.035**	0.635	1.887	0.768-4.634	0.166
BCLC stage (C vs. B vs. A)	0.440	1.553	1.053-2.292	**0.027**	0.036	1.037	0.652-1.648	0.878
Child-Pugh classification grade (B vs. A)	1.319	3.738	1.286-10.865	**0.015**	0.432	1.540	0.428-5.545	0.509
Microvascular invasion (positive vs. negative)	1.181	3.256	1.446-7.332	**0.004**	0.979	2.661	1.102-6.427	**0.030**
Edmondson-Steiner grade (III-IV vs. I-II)	0.708	2.030	0.948-4.346	**0.068**	0.933	2.543	1.069-6.048	**0.035**
FAM99A expression (high vs. low)	-0.960	0.383	0.179-0.820	**0.013**	-0.855	0.425	0.189-0.958	**0.039**

Statistically significant *P* values are in bold (*P* < 0.10)

*HCC* hepatocellular carcinoma; *AFP* alpha-fetoprotein; *BCLC* Barcelona Clinic Liver Cancer; *HR* hazard ratio; *CI* confidence interval

### FAM99A suppressed HCC cell viability and clonogenicity in vitro

**Fig. 4:**
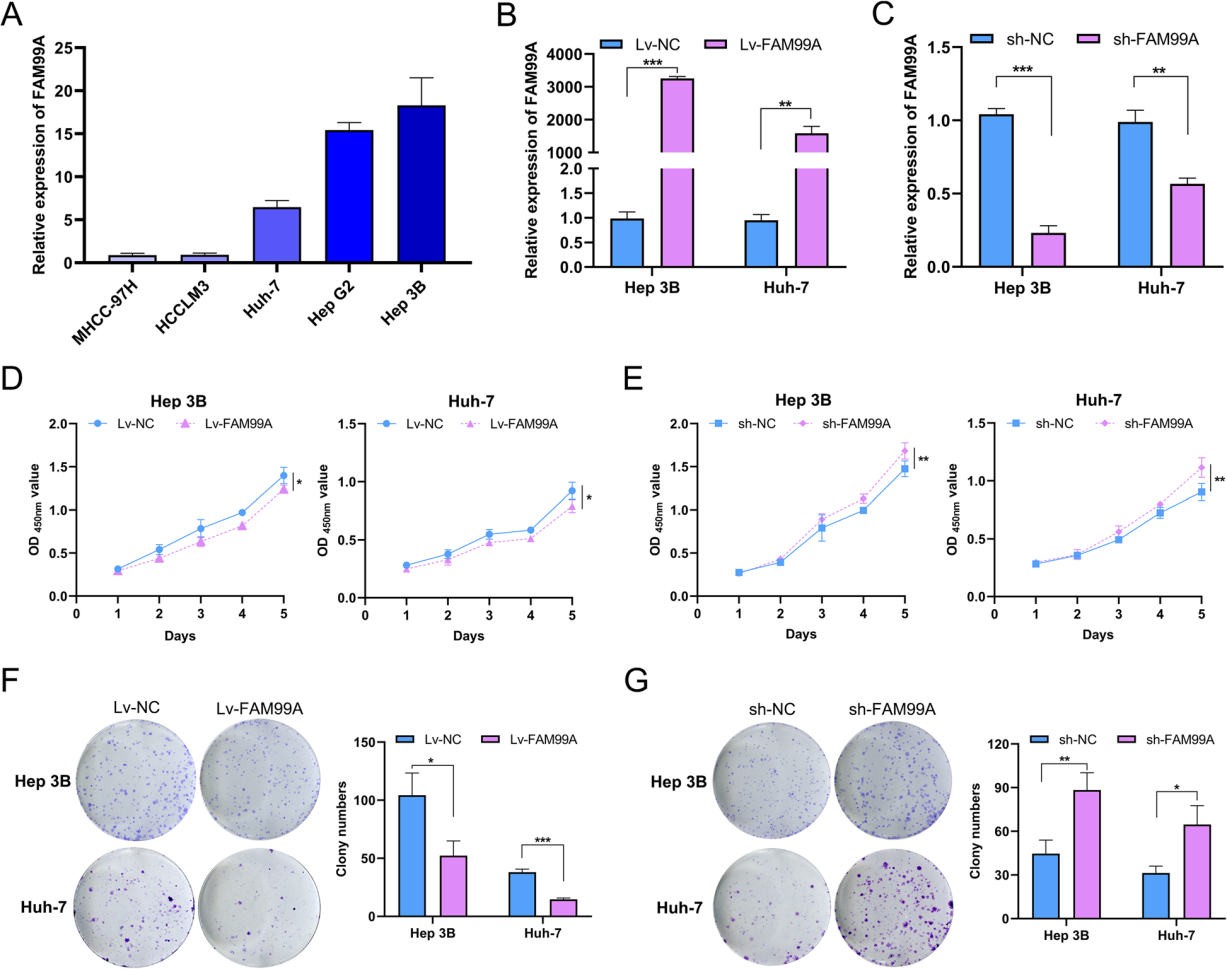
FAM99A suppresses the proliferation ability of HCC cells in vitro. **A** Relative expression level of FAM99A in five HCC cell lines. **B** The overexpression efficiency of Lv-FAM99A was examined using qRT-PCR in Hep 3B and Huh-7 cells. **C** The knockdown efficiency of sh-FAM99A was examined using qRT-PCR in Hep 3B and Huh-7 cells. **D** CCK-8 assay showed that upregulation of FAM99A inhibited the proliferation of Hep 3B and Huh-7 cells. **E** CCK-8 assay indicated that downregulated FAM99A promoted the proliferation of Hep 3B and Huh-7 cells. **F** Colony formation assays showed that overexpressing FAM99A reduced the number of colonies. **G** Colony formation assays revealed that knocking down of FAM99A increased the number of colonies. All experiments were repeated three times. *P < 0.05; **P < 0.01; ***P < 0.001

To determine the function of FAM99A in HCC cell lines, qRT-PCR was performed to examine the expression level of FAM99A in six HCC cell lines, and the results are shown in Fig. 4A. Hep 3B and Huh-7 cells with relatively high expression of FAM99A were selected for gain-of-function and loss-of-function analyses. The qRT-PCR results showed that FAM99A was successfully overexpressed in the Lv-FAM99A group in Hep 3B (*P* < 0.001) and Huh-7 cells (*P* < 0.01; Fig. 4B) but was downregulated in the sh-FAM99A group in Hep 3B (*P* < 0.001) and Huh-7 cells (*P* < 0.01; Fig. 4C) compared to the corresponding negative control group.

The CCK-8 assay showed that the overexpression of FAM99A significantly inhibited the viability of Hep 3B and Huh-7 cells (both *Ps* < 0.05; Fig. 4D). In contrast, FAM99A knockdown evidently facilitated the viability of Hep 3B and Huh-7 cells (both *Ps* < 0.01; Fig. 4E). The colony formation assay also showed that overexpression of FAM99A remarkably impeded the clonogenicity of Hep 3B (*P* < 0.05) and Huh-7 cells (*P* < 0.001; Fig. 4F), but the colonies were significantly increased when FAM99A was knocked down in Hep 3B (*P* < 0.01) and Huh-7 cells (*P* < 0.05; Fig. 4G). These results indicated that FAM99A suppressed the proliferation of HCC cells.

### FAM99A impeded the migration and invasion capacities of HCC cells in vitro

**Fig. 5:**
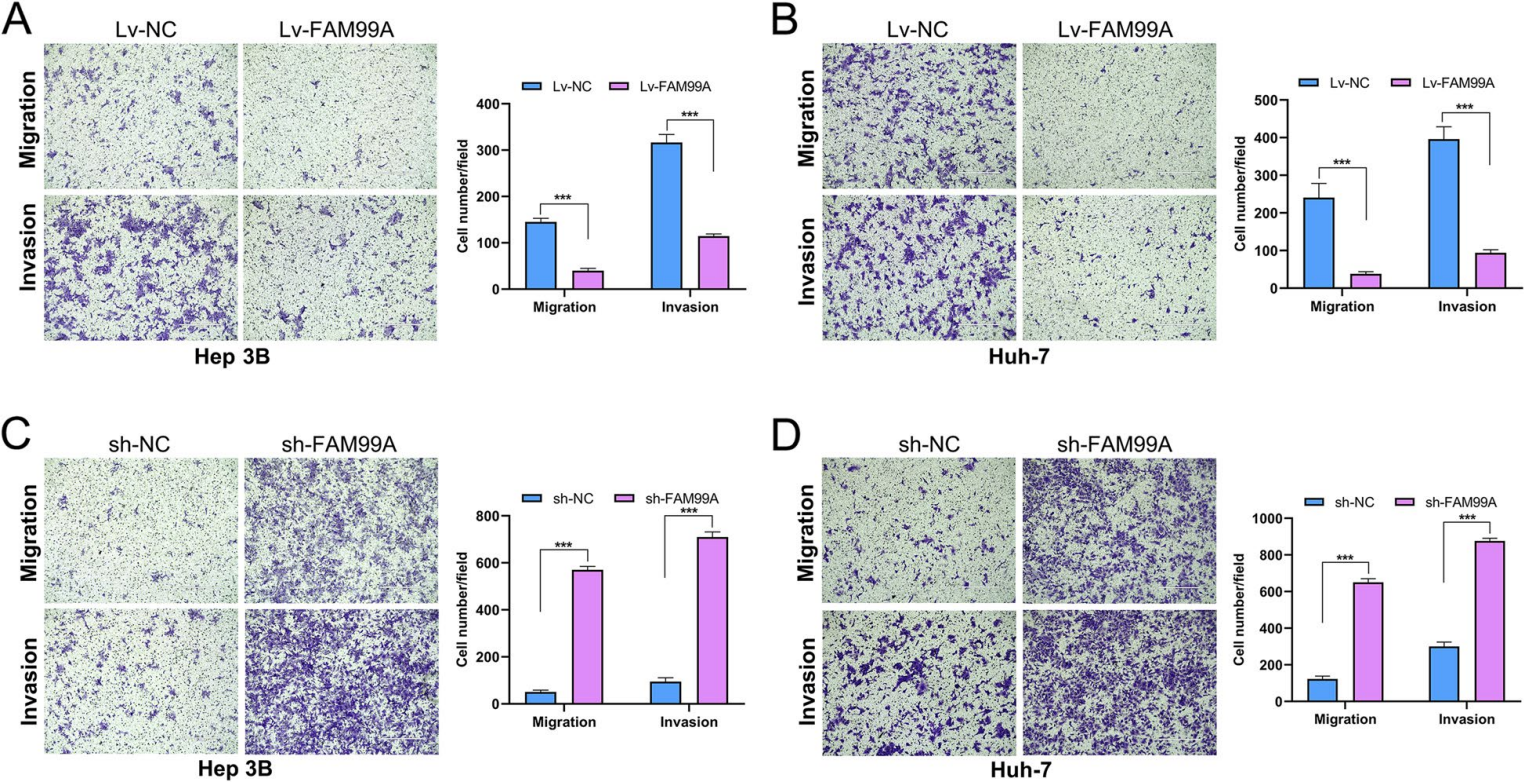
FAM99A impedes the migration and invasion activities of HCC cells in vitro. **A**, **B** Overexpression of FAM99A suppressed the migration and invasion abilities of Hep-3B and Huh-7 cells. Scale bar: 400 μm. **C**, **D** FAM99A knockdown strengthened the migration and invasion abilities of Hep-3B and Huh-7 cells. Scale bar: 400 μm. ***P < 0.001.

The effects of FAM99A on the migration and invasion of HCC cells were further investigated using Transwell migration and invasion assays. Upregulation of FAM99A significantly restrained the cell migration and invasion abilities of Hep 3B and Huh-7 cells (all *Ps* < 0.001; Fig. 5A, B). In contrast, silencing FAM99A remarkably enhanced the cell migration and invasion activities of Hep 3B and Huh-7 cells (all *Ps* < 0.001; Fig. 5C, D). These findings suggested that FAM99A inhibited the migration and invasion behavior of HCC cells.

### FAM99A inhibited HCC cell tumor growth in vivo

**Fig. 6:**
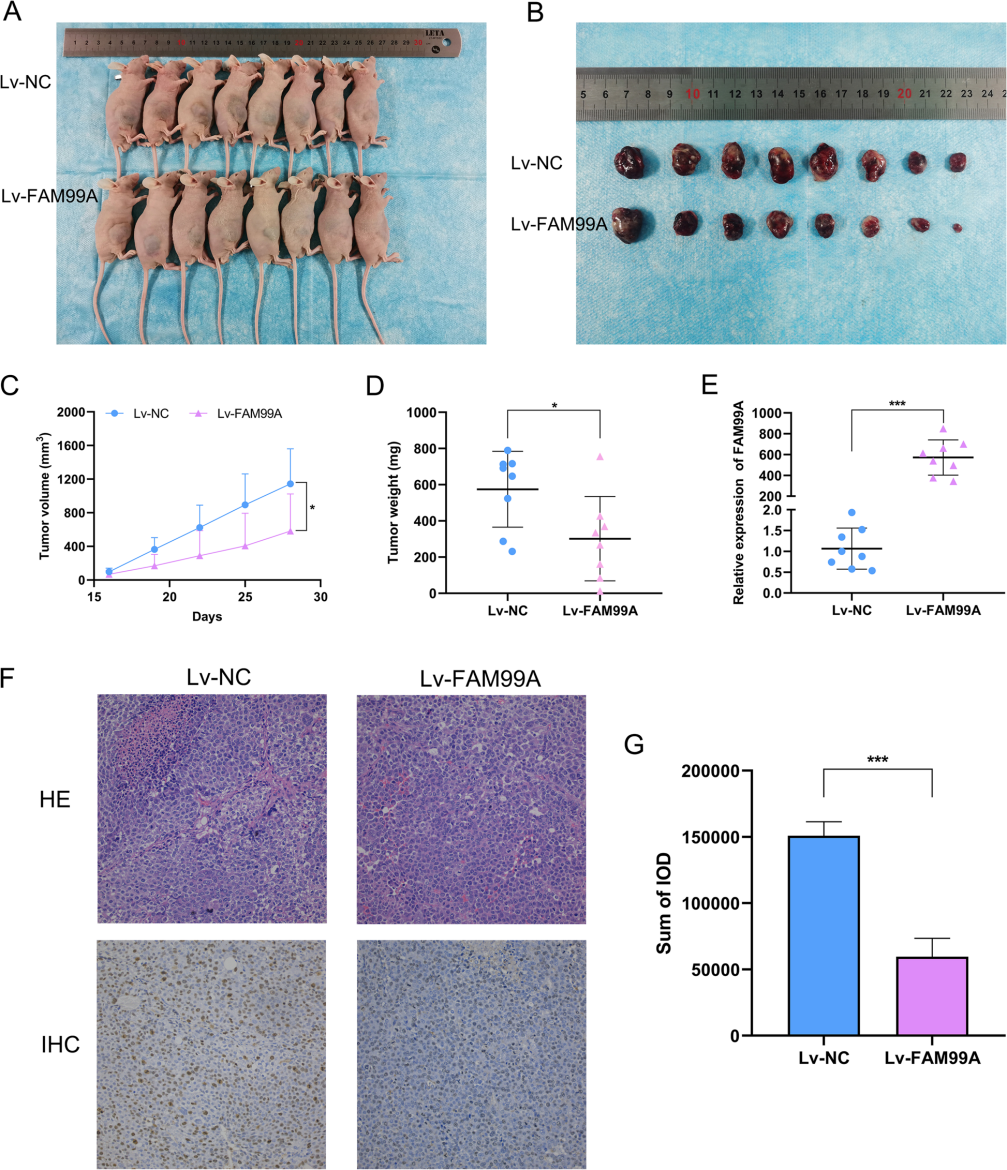
FAM99A inhibits the tumor growth of HCC cells in vivo. **A**, **B** Construction of the subcutaneous xenograft tumor model in nude mice. **C** The growth curves of tumor volume in nude mice with negative control and FAM99A overexpression. **D** The weights of subcutaneous tumors in nude mice with negative control and FAM99A overexpression. **E** Relative expression levels of FAM99A in subcutaneous tumors were verified using qRT-PCR. **F** Representative images of hematoxylin-eosin (HE) staining and Ki-67 immunohistochemical (IHC) staining from the tumor sections. **G** Sum of integrated optical density (IOD) of Ki-67 staining in the tumor sections. *P < 0.05; ***P < 0.001.

To test whether FAM99A suppressed tumor growth in vivo, a subcutaneous xenograft tumor model was constructed in nude mice (Fig. 6A, B). As shown in Fig. 6C and D, the tumor volume and tumor weight of the Lv-FAM99A group were lower than the control group, which demonstrated that FAM99A markedly impeded the tumor growth of Huh-7 cells in vivo (both *Ps* < 0.05). The FAM99A expression level in solid tumors was confirmed using qRT-PCR, and the results revealed that FAM99A expression was significantly higher in the Lv-FAM99A group than the control group (*P* < 0.001; Fig. 6E). HE staining suggested that upregulation of FAM99A decreased cell necrosis and infiltration of tumor tissues compared to the negative control group (Fig. 6F). The staining intensity of the proliferation marker Ki-67 in the Lv-FAM99A group was evidently weakened compared to the control group (*P* < 0.001; Fig. 6G). These results confirmed that FAM99A suppressed HCC cell growth in vivo.

### FAM99A may interact with seven important proteins to regulate HCC progression

**Fig. 7:**
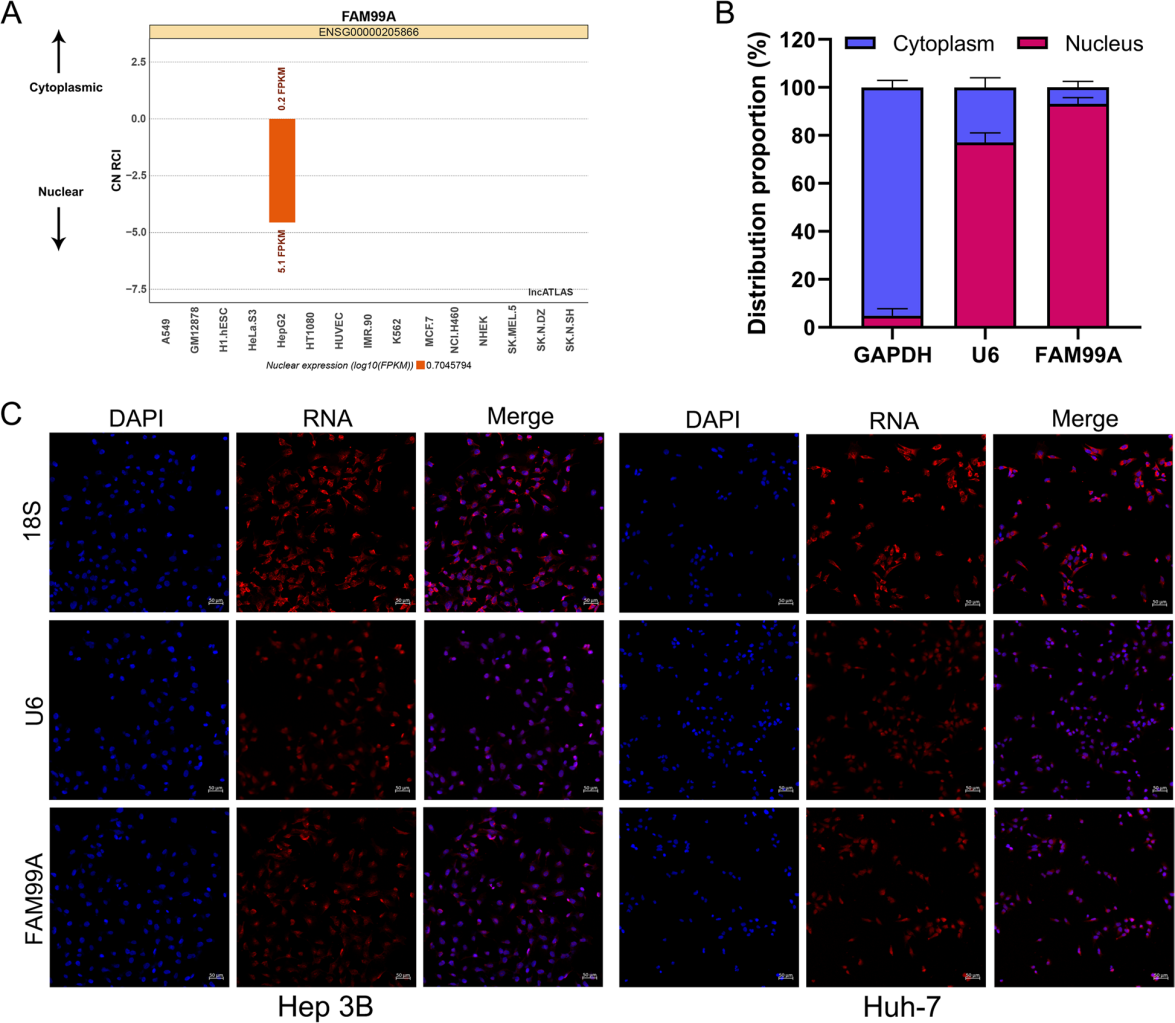
The subcellular localization of FAM99A. **A** The subcellular localization of FAM99A in human cells based on the lncATLAS website (https://lncatlas.crg.eu/). **B** Relative distribution proportion of FAM99A in nuclear and cytoplasmic fractions in Hep 3B cells was determined using qRT-PCR. **C** RNA-FISH assay showed the enrichment of FAM99A in the nuclei of Hep 3B and Huh-7 cells. Scale bar: 50 μm.

The subcellular localization of lncRNAs determines the dominant mechanism of its molecular functions. We examined the subcellular localization of FAM99A using the lncATLAS website, which is a comprehensive resource of lncRNA localization in human cells based on RNA-seq datasets [23] (https://lncatlas.crg.eu/). The results suggested that FAM99A was primarily located in the nuclei of HepG2 cells and showed a characteristic liver-specific expression pattern (Fig. 7A). Subcellular fractionation and FISH assays were performed to confirm the results from the online database. Subcellular fractionation analysis indicated that FAM99A was mostly located in the nucleus of Hep 3B cells (93.16% in the nucleus vs. 6.84% in the cytoplasm; Fig. 7B). As shown in Fig. 7C, the FISH assay also suggested that FAM99A was primarily localized in the nuclei of Hep 3B and Huh-7 cells.

**Fig. 8:**
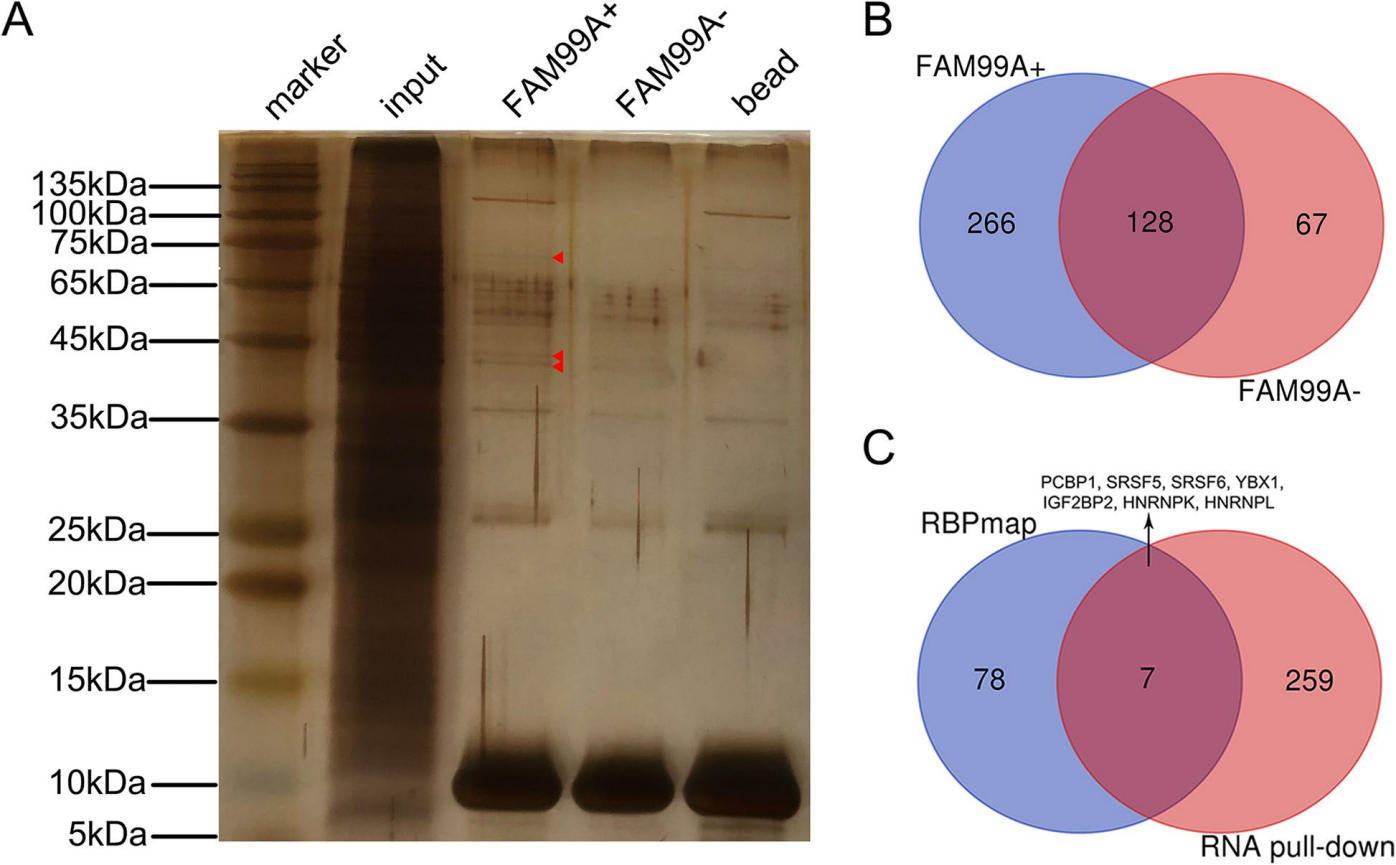
The RNA pull-down assay and mass spectrometry analysis of FAM99A. **A** Silver staining of the indicated electrophoretic bands of proteins pulled down by biotin-labeled FAM99A in Hep 3B cells. **B** Venn diagram of the proteins pulled down by FAM99A (FAM99A+) and antisense FAM99A (FAM99A-). There were 266 differential proteins only identified by FAM99A+. **C** Venn diagram of the potential binding proteins of FAM99A identified using the RNA pull-down assay and the predicted result of the RBPmap website (http://rbpmap.technion.ac.il/)

An RNA pull-down assay was performed to identify proteins that may combine with FAM99A, and mass spectrometry analysis identified 266 differential proteins of FAM99A compared with its antisense strand (Additional file 2: Table S1; Fig. 8A, B). These 266 differential proteins were imported into DAVID 6.8 [24] (https://david.ncifcrf.gov/), and Kyoto Encyclopedia of Genes and Genomes (KEGG) pathway enrichment [25–27] was performed. The results suggested that these proteins were primarily enriched in the “Biosynthesis of antibiotics”, “Carbon metabolism”, “Ribosome”, “Complement and coagulation cascades”, “Biosynthesis of amino acids”, “Pentose phosphate pathway”, “Glycolysis/Gluconeogenesis”, and “Focal adhesion” pathways (Benjamin adjust *P* < 0.05; Table 5). We also predicted the RNA binding proteins of FAM99A using the RBPmap website [28] (http://rbpmap.technion.ac.il/). We found seven proteins that were recognized in the RNA pull-down and RBPmap results, including PCBP1 (Poly (rC) binding protein 1), SRSF5 (serine/arginine-rich splicing factor 5), SRSF6 (serine/arginine-rich splicing factor 6), YBX1 (Y-box-binding protein 1), IGF2BP2 (insulin-like growth factor 2 mRNA-binding protein 2), HNRNPK (heterogeneous nuclear ribonucleoprotein K), and HNRNPL (heterogeneous nuclear ribonucleoprotein L) (Fig. 8C).

**Table 5:** Significant KEGG pathways of 266 RNA-pull down proteins of FAM99A

Category	Term	Genes	%	***P***-Value	Benjamini
KEGG_PATHWAY	Biosynthesis of antibiotics	19	7.3	1.20E-06	1.10E-04
KEGG_PATHWAY	Carbon metabolism	14	5.3	1.40E-06	1.10E-04
KEGG_PATHWAY	Ribosome	15	5.7	2.10E-06	1.10E-04
KEGG_PATHWAY	Complement and coagulation cascades	10	3.8	2.20E-05	8.90E-04
KEGG_PATHWAY	Biosynthesis of amino acids	10	3.8	3.20E-05	1.00E-03
KEGG_PATHWAY	Pentose phosphate pathway	6	2.3	4.30E-04	1.10E-02
KEGG_PATHWAY	Glycolysis / Gluconeogenesis	8	3.1	7.50E-04	1.50E-02
KEGG_PATHWAY	Focal adhesion	14	5.3	7.60E-04	1.50E-02

*KEGG* kyoto encyclopedia of genes and genomes

## Discussion

Liver-specific lncRNAs are emerging as pivotal regulators in the tumorigenesis and progression of HCC. We identified for the first time that FAM99A exhibited a highly liver-specific expression pattern, which suggests that FAM99A plays important roles in HCC. FAM99A was steadily downregulated in HCC tissues and negatively correlated with vascular invasion and advanced histological grade of HCC patients. The Kaplan-Meier curve analysis also revealed that patients with low expression of FAM99A tended to have poor OS based on the data from the TCGA database, ICGC database, and our HCC cohort. A multivariate Cox regression model demonstrated that FAM99A was an independent factor for the OS of HCC patients. In vitro experiments indicated that overexpression of FAM99A inhibited the cell proliferation, migration, and invasion abilities of HCC cell lines, and knocking down FAM99A produced the opposite effects. The subcutaneous tumor formation model suggested that FAM99A suppressed tumor growth of HCC cells in vivo. Our findings suggested that FAM99A exerted a cancer-inhibiting effect in HCC progression.

Patients with malignant clinical features tend to have a poor prognosis in HCC. Our study found that patients with reduced expression of FAM99A were more likely to develop microvascular invasion and advanced histological grade. Another study also revealed that the downregulation of FAM99A was significantly associated with incomplete tumor capsule, tumor differentiation, recurrence, and poor prognosis of HCC patients [29]. These two studies revealed that FAM99A was an independent prognostic indicator for the OS of HCC patients. These results suggested that decreased expression of FAM99A contributed to the progression of HCC and may be a promising prognostic indicator for HCC patients.

To the best of our knowledge, we are the first to identify FAM99A as a liver-specific lncRNA based on the GTEx project. A series of gain- and loss-of-function studies suggested that FAM99A suppressed the proliferation, migration, and invasion of HCC cells. Recent studies also reported the antitumorigenic functions of FAM99A in HCC. BX Zhao et al. found that FAM99A inhibited HCC metastasis and epithelial-mesenchymal transition by sponging miR-92a [29]. Another study demonstrated that FAM99A suppressed HCC cell viability and GLUT1-mediated glycolysis, which inhibited HCC progression [30]. Many abnormally expressed tissue-specific lncRNAs are involved in the tumorigenesis and progression of cancers. LINC00993 is a breast-specific lncRNA that is downregulated in triple-negative breast cancer (TNBC), and it suppressed the tumor growth of TBNC [31]. Testis developmental related 1 (TDRG1), also known as LINC00532, is expressed exclusively in the testis. TDRG1 is upregulated in testicular seminoma tissues and promotes the development, migration, and chemotherapy resistance of seminoma cells [32–34]. Prostate enriched lncRNA (PSLNR) is a prostate-specific lncRNA that inhibits prostate cancer progression via the p53-dependent pathway [35]. In contrast, another two prostate-specific lncRNAs (PCGEM1 and PCA3) are overexpressed in prostate cancer and promote the cell proliferation ability and inhibit the cell apoptosis ability of prostate cancer cells [36–39]. Notably, PCA3 is promising as a more efficiency diagnostic biomarker for prostate cancer than the currently used prostate-specific antigen [40, 41]. Given the important roles of tissue-specific lncRNAs in cancer, more studies are needed to further explore FAM99A as a diagnostic biomarker or therapeutic target for HCC patients.

The molecular mechanisms of lncRNAs primarily depend on its localization in cells. We revealed for the first time that FAM99A was primarily located in the nucleus using subcellular fractionation and RNA-FISH assays, which indicated that FAM99A may play key regulatory roles in pivotal nuclear processes, such as chromatin organization, transcriptional and post-transcriptional programs, subcellular structures, and nuclear structure organization. These nuclear processes required the interaction of lncRNAs with RNA binding proteins (RBPs) almost universally [42, 43]. Therefore, we performed an RNA pull-down assay and identified that FAM99A pulled down 266 proteins using mass spectrometry analysis. To reveal the underlying mechanisms of FAM99A in HCC, these 266 proteins were used in KEGG pathway enrichment analysis. The results demonstrated that these proteins were primarily involved in several important cancer-related pathways, including complement and coagulation cascades [44], the pentose phosphate pathway [45–47], glycolysis/gluconeogenesis [48], and focal adhesion [49, 50]. We also identified seven proteins (PCBP1, SRSF5, SRSF6, YBX1, IGF2BP2, HNRNPK, and HNRNPL) that may interact with FAM99A. These seven proteins play crucial roles in tumor occurrence and development. PCBP1 is a multifunctional RBP that regulates the alternative splicing, translation, and RNA stability of many cancer-related genes to exert its cancer-inhibiting effect [51–53]. SRSF5 and SRSF6 belong to the serine/arginine-rich (SR) protein family, which is an important class of splicing regulators. SRSF5 and SRSF6 play important roles in the development and progression of cancers [54–57]. YBX1, also known as YB-1, binds DNA and RNA, and it is closely related to various malignant phenotypes of cancer cells, including tumor cell proliferation, metastasis, angiogenesis, and drug resistance [58, 59]. IGF2BP2 is a member of the conserved oncofetal RNA-binding protein family that acts as a N6-methyladenosine (m6A) reader, and it is involved in the development and progression of various cancer types [60–62]. HNRNPK and HNRNPL belong to the heterogeneous nuclear ribonucleoprotein family and interact with tumor-associated lncRNAs to regulate the tumorigenesis and progression of various cancers, including HCC [63–65]. Given the great importance of these seven proteins, further validation and functional experiments are needed to reveal the relationships between FAM99A and these proteins.

To the best of our knowledge, the current study is the first study to fully examine the expression level and clinical and prognostic significance of FAM99A in HCC based on three public online databases and our own HCC cohort. The current study used meta-analysis to pool FAM99A expression in HCC based on 18 GEO datasets for the first time. The ICGC database was used for the first time to examine the expression level and survival significance of FAM99A in HCC. We also determined the subcellular localization of FAM99A in HCC cells for the first time and performed an RNA pull-down assay to enrich the mechanistic research of FAM99A in HCC development. Although we identified seven key RBPs that may interact with FAM99A, we were unable to verify their binding modes and mechanisms due to limitations of the experimental conditions and experimental levels. Therefore, further in-depth studies are needed to elucidate the comprehensive mechanisms of FAM99A in HCC development.

## Conclusion

FAM99A is a liver-specific lncRNA that is downregulated in HCC and negatively associated with poor prognosis in HCC patients. It may exert its tumor-suppressing function via binding with some critical RBPs. Our study enriches the understanding of the effects of FAM99A in HCC and suggests that it may serve as a promising prognostic biomarker for HCC patients.

## Supplementary Information

**Additional file 1.**

**Additional file 2.**

**Additional file 3.**

## Data Availability

Access to public data is described in the manuscript. Experimental data of our study is available from the corresponding author on reasonable request.

## Abbreviations

lncRNAs: Long non-coding RNAs
HCC: Hepatocellular carcinoma
TCGA: The Cancer Genome Atlas
ICGC: International Cancer Genome Consortium
GEO: Gene Expression Omnibus
qRT–PCR: Quantitative real-time polymerase chain reaction
PSTAR: p53-stabilizing and activating RNA
HULC: Highly upregulated in liver cancer
GTEx: Genotype-Tissue Expression
RNA-seq: RNA sequencing
TPM: Transcripts per million
OS: overall survival
DMEM: Dulbecco’s modified Eagle’s medium
MEM: Minimum Essential Medium
FBS: Fetal bovine serum
CCK-8: Cell Counting Kit-8
SPF: Specific pathogen free
HE: Hematoxylin-eosin
IHC: Immunohistochemical
FISH: Fluorescence in situ hybridization
DAPI: 4′-6-diamidino-2-phenylindole
SD: Standard deviation
SMD: Standardized mean difference
CI: Confidence interval
KEGG: Kyoto Encyclopedia of Genes and Genomes
PCBP1: Poly(rC) binding protein 1
SRSF5: Serine/arginine-rich splicing factor 5
SRSF6: Serine/arginine-rich splicing factor 6
YBX1: Y-box-binding protein 1
IGF2BP2: Insulin-like growth factor 2 mRNA-binding protein 2
HNRNPK: Heterogeneous nuclear ribonucleoprotein K
HNRNPL: Heterogeneous nuclear ribonucleoprotein L
TNBC: Triple-negative breast cancer
RBPs: RNA binding proteins
m6A: N6-methyladenosine
