# Supplementary figures and images for: Liver-specific lncRNA FAM99A may be a tumor suppressor and promising prognostic biomarker in hepatocellular carcinoma
Source: BMC Cancer. 2022 Oct 26;22:1098. doi: 10.1186/s12885-022-10186-2 (PMC9609286; doi:10.1186/s12885-022-10186-2)

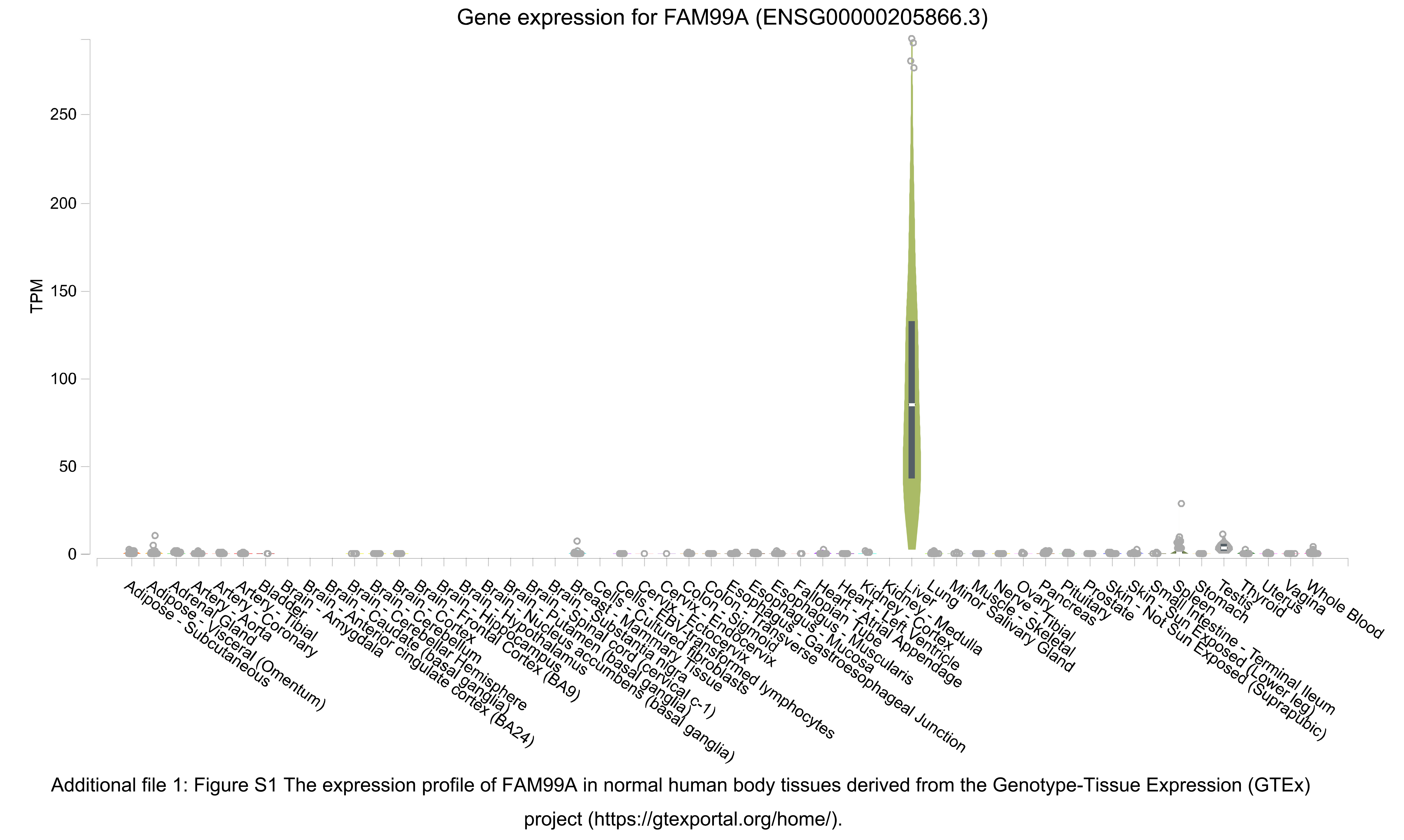

Supplement: Supplementary file 1 — Additional file 1. [file 12885_2022_10186_MOESM1_ESM.tif]

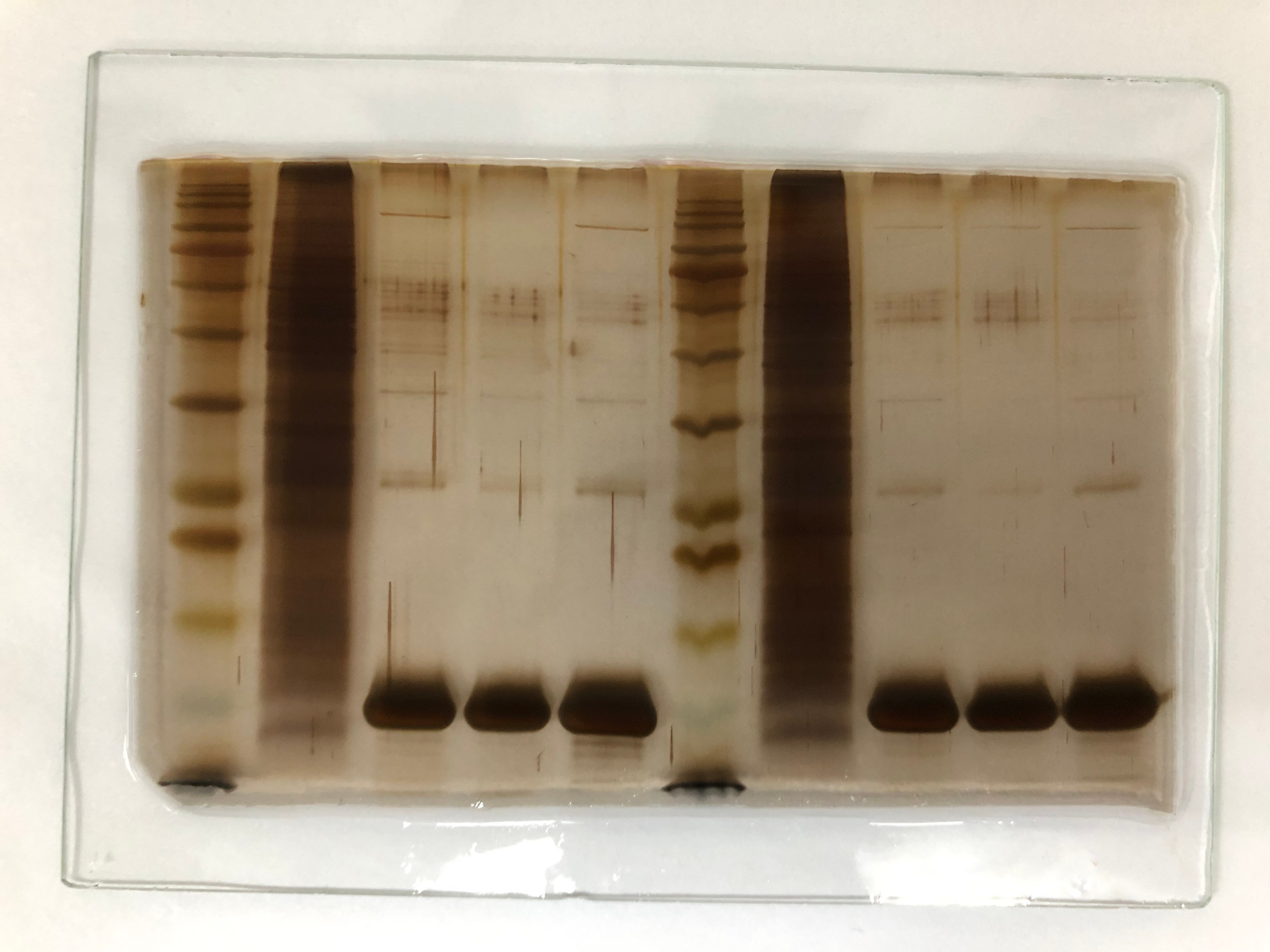

Supplement: Supplementary file 3 — Additional file 3. [file 12885_2022_10186_MOESM3_ESM.jpg]
